# Identification and analysis of the RNA degrading complexes and machinery of *Giardia lamblia *using an *in silico *approach

**DOI:** 10.1186/1471-2164-12-586

**Published:** 2011-11-29

**Authors:** Christopher W Williams, Heidi G Elmendorf

**Affiliations:** 1 Department of Biology, Georgetown University, Washington, DC 20057, USA

## Abstract

**Background:**

RNA degradation is critical to the survival of all cells. With increasing evidence for pervasive transcription in cells, RNA degradation has gained recognition as a means of regulating gene expression. Yet, RNA degradation machinery has been studied extensively in only a few eukaryotic organisms, including *Saccharomyces cerevisiae *and humans. *Giardia lamblia *is a parasitic protist with unusual genomic traits: it is binucleated and tetraploid, has a very compact genome, displays a theme of genomic minimalism with cellular machinery commonly comprised of a reduced number of protein components, and has a remarkably large population of long, stable, noncoding, antisense RNAs.

**Results:**

Here we use *in silico *approaches to investigate the major RNA degradation machinery in *Giardia lamblia *and compare it to a broad array of other parasitic protists. We have found key constituents of the deadenylation and decapping machinery and of the 5'-3' RNA degradation pathway. We have similarly found that all of the major 3'-5' RNA degradation pathways are present in *Giardia*, including both exosome-dependent and exosome-independent machinery. However, we observe significant loss of RNA degradation machinery genes that will result in important differences in the protein composition, and potentially functionality, of the various RNA degradation pathways. This is most apparent in the exosome, the central mediator of 3'-5' degradation, which apparently contains an altered core configuration in both *Giardia *and *Plasmodium*, with only four, instead of the canonical six, distinct subunits. Additionally the exosome in *Giardia *is missing both the Rrp6, Nab3, and Nrd1 proteins, known to be key regulators of noncoding transcript stability in other cells.

**Conclusions:**

These findings suggest that although the full complement of the major RNA degradation mechanisms were present - and likely functional - early in eukaryotic evolution, the composition and function of the complexes is more variable than previously appreciated. We suggest that the missing components of the exosome complex provide an explanation for the stable abundance of sterile RNA species in *Giardia*.

## Background

Cells control RNA levels through the regulation of both transcription and degradation. Organisms must degrade not only aberrantly folded or misprocessed RNAs, but also functional RNA transcripts that are no longer needed by the cell. In order to distinguish among and degrade only the appropriate RNA transcripts, cells have developed multiple RNA degradation processes and complexes. RNA degradation can occur by digestion inwards from the ends, using 5' to 3' and 3' to 5' exonucleases, or by digestion at internal sites using endonucleases. RNA degradation often, though not always, also involves deadenylation (for both 5' to 3' and 3' to 5' degradation) and decapping (for 5' to 3' degradation) of the transcripts. The range of RNA degradation machinery includes both nuclear and cytoplasmic components.

RNA degradation typically begins with the shortening of the long poly-A tail of mRNA transcripts. Although some RNA degradation pathways can apparently act on polyadenylated transcripts, e.g. nonstop decay (NSD), nonsense-mediated decay (NMD), and endonucleolytic cleavage (RNAi), the majority of 5' to 3' and 3' to 5' exonucleolytic activities require the prior removal of the poly-A tail. Two proteins, Ccr4p and/or Caf1p, have been shown to act as the catalytic core of the deadenylation machinery in all eukaryotic cells examined to date [1-3]. 5' to 3' degradation depends on the prior removal of the 5' cap structure followed by subsequent 5' to 3' exonucleolytic cleavage. In most cells this is performed by the Dcp1p/Dcp2p holoenzyme with the involvement of a wide and diverse array of additional protein machinery [4-6].

The exosome, a 3' to 5' exonuclease complex, is one of the important RNA degradation complexes and can be used as a means of classifying the different machinery into two groups: the exosome-dependent and the exosome-independent pathways. It is this convention that we use here to describe the RNA degradation machinery in *Giardia lamblia *and other parasitic protists. Exosomes have clear homologs in all three domains of life. Bacteria, archaea, and eukaryotes possess functionally analogous core 3' to 5' RNA degradation complexes in the bacterial polynucleotide phosphorylase (PNPase), the archaeal exosome, and the eukaryotic exosome, respectively. The similarity in the structure of all three mRNA degradation complexes is striking and suggests that the highly conserved structures are necessary for mRNA degradation and have been maintained throughout evolutionary history.

The bacterial PNPase exists as a homotrimer, in which each monomer possesses two tandem RNase PH domains in addition to single S1 and KH domains [7]. RNase PH domains have exonucleolytic activity, although only one of the two domains in each monomer is thought to be active, while the S1 and KH domains have RNA binding capacity [7]. The archaeal exosome ring is composed of repeating Rrp41/42 heterodimers arranged into a hexamer with three total copies of the stabilizing proteins Rrp4 and Csl4 acting as caps to the complex. The Rrp41 and Rrp42 subunits possess RNase PH domains, but Rrp41 is the only exonucleolytic component of the complex, again resulting in three active sites per complex [7,8]. Rrp4 and Csl4 both possess S1 domains and bind RNA.

In eukaryotes, the core exosome also exists as a ring structure made of a heterohexamer of proteins with RNase PH domains (the three Rrp 41-like proteins are Rrps41, 46 and Mtr3, and the three Rrp 42-like proteins are Rrps42, 43, and 45) with a trio of additional RNA-binding proteins which contain S1 domains (Rrps 4, 40, and Csl4) that broadly act as the entry to the pore of the exosome and in eukaryotic exosomes further act to stabilize the hexameric ring [9,10]. It is believed that, through gene duplication either early in the eukaryotic lineage or prior to the divergence of eukaryotes, *rrp41 *gave rise to both *rrp46 *and *mtr3*, while *rrp42 *gave rise to *rrp43 *and *rrp45 *[7]. The ring and stabilizing proteins are commonly associated with Rrp6 and Rrp44, both of which possess nucleolytic activity [9,11]. The core proteins display homology to archaeal exosome and bacterial PNPase proteins, whereas Rrp6 and Rrp44 display homology to bacterial RNases [12-14]. In some eukaryotes, the RNase PH domain of Rrp41 provides exonucleolytic activity, whereas in other species the activity is dependent upon Rrp6 and Rrp44 [9,11,15].

Although the exosome is an important complex involved in RNA degradation in eukaryotes, additional complexes also play a role in RNA degradation either through exosome-dependent or exosome-independent processes. Exosome-dependent complexes act mainly by preparing RNA substrates for degradation by the exosome, whereas exosome-independent complexes possess nucleolytic activity of their own. These additional complexes impart specificity to its function so that RNA is not degraded prematurely, and only a subset of RNA is targeted at any one time.

The exosome-dependent machinery includes the TRAMP complex, Pumilio (Puf) proteins, Nonsense mediated decay (NMD), Nonstop decay (NSD), and No-go decay (NGD) complexes. In the nucleus, the exosome can be found to be associated with the TRAMP complex, which aids in the degradation, maturation, and removal of secondary structures of RNA molecules through the post-transcriptional addition of a poly-A tail by TRAMP proteins Trf4/5 or via the helicase activity of TRAMP protein Mtr4, respectively [16,17]. In the cytoplasm a subset of Puf proteins bind mRNAs via sequence-specific elements in the 3' untranslated regions (UTRs) and recruit the deadenylation machinery [18,19]. Also in the cytoplasm, the NMD, NSD, and NGD pathways act as mRNA quality control and are activated in response to mRNAs containing premature termination codons (PTCs), no stop codon, or secondary structures such as stem loops, respectively. The NMD complex may possess endonucleolytic activity, but requires the exosome for complete degradation of RNAs.

The exosome-independent complexes are the RNAi machinery and the Ccr4-Not complex. RNAi acts to silence gene expression through endonucleolytic degradation of targeted mRNA transcripts or translation inhibition. RNAi machinery has been identified in a variety of eukaryotic organisms, from single-celled organisms to metazoans but is not ubiquitously present. Two components of the Ccr4-Not complex, which is conserved from *S. cerevisiae *to humans [20], have roles in mRNA deadenylation; Ccr4 and Caf1 deadenylate mRNA transcripts, although optimal degradation for many transcripts still requires the exosome [21].

The parasitic protists regulate gene expression through many different mechanisms, both transcriptional and post-transcriptional. Yet, while we understand much about transcriptional regulation, the study of mRNA degradation machinery in the parasitic protists is still in its early stages. This is perhaps especially surprising because RNA degradation is likely to play an unusually prominent role in organisms that exhibit diminished regulation of gene expression at the transcriptional level, as is known to be the case for several parasitic protists. For example, *Trypanosoma brucei *transcribes its genes polycistronically, implying that mRNA processing and degradation are its primary means of regulating gene expression [22-24]. And *Giardia lamblia *transcribes an abundance of full-length sterile antisense transcripts that are capped and polyadenylated [25], suggesting a role for mRNA degradation to eliminate these aberrant transcripts.

In this paper, we discuss our efforts to identify the mRNA degradation machinery in *Giardia lamblia *using *in silico *approaches. We additionally included several other parasitic protists (*Entamoeba histolytica*, *Trichomonas vaginalis*, *Trypansoma brucei*, *and Plasmodium falciparum*) in our analyses of the core and peripheral exosome components for comparison, building on the work of previous researchers in this field [1,23,24,26-33]. We focused especially on *Giardia *given its evolutionary divergence [34,35], severely reduced repertoire of transcriptional machinery [34,36], and unusual patterns of gene expression [37,38]. We identified an extensive collection of genes coding for proteins with significant sequence similarity to proteins that participate in RNA degradation pathways in other eukaryotes. Pathways such as the RNAi [39-41] and nonsense mediated decay pathways [28,42] previously have been identified in *Giardia*. However, these comparisons also revealed that a substantial number of protein constituents of mRNA degradation complexes in other eukaryotes are either absent or sufficiently divergent to thwart detection by similarity searches in these parasitic protists. We use this new knowledge to consider which protein components may comprise the most reduced core exosome structure in eukaryotes and to postulate explanations for observed patterns of mRNA transcripts in *Giardia*.

## Results

### Preparing the transcripts for degradation

#### Deadenylation machinery

Deadenylation, the removal of the 3' polyA tail, is typically performed by Ccr4p and Caf1p of the Ccr4-Not complex found in most eukaryotes studied to date, while the Pan2p/Pan3p complex plays a secondary role [1,3]. The Ccr4-Not complex is a multi-subunit complex with roles in transcription, mRNA and protein degradation, and cell division [20]. Members of the complex are the Not proteins 1-5, Ccr4, and the Caf proteins, Caf1 (also known as Pop2), Caf40, and Caf130. The Not1 protein acts as a scaffold to which other members of the complex attach and is necessary for *S. cerevisiae *viability [20]. Pop2p acts a scaffold for Ccr4p and also may have additional roles in decapping of transcripts in *S. cerevisiae *[2].

*Giardia *possesses only a subset of the genes associated with the Ccr4-Not complex that may be sufficient for its functionality in the parasite (Additional Files 1 and 2). We identified four candidate *Not *genes, as well as candidate genes for *Caf1*, confirming a previous identification of *Caf1 *in *Giardia *[1]. The classification of three of the *Not *genes as *Not1*, *Not2*, and *Not4 *was possible based on the presence of defined domains. The protein identified as most similar to Not1p has only a partial Not1 domain, while Not2p and Not4p possess complete domains. The fourth protein could possibly be either Not3p or Not5p based on sequence similarity search results, although the better scores and E-values obtained against Not3 proteins suggests it is more likely Not3p. Although we were unable to detect homologs of *Ccr4, Caf40, Caf130 *or *CNot10 *in the *Giardia *genome, the presence of the Not proteins and Caf1p should be sufficient to ensure a functional deadenylation complex.

The Pan2-Pan3 complex is thought to play a role in either initiating or subsequently trimming the poly-A tails in support of Ccr4-Caf1-Not complex activity. The complex functions through association of Pan3 with Pabp1 to recognize and associate with the poly-A tail of transcripts. We were able to detect genes with sequence similarity to *Pan2 *and *Pabp1 *in *Giardia *but could not detect a *Pan3 *homolog, raising questions about whether this complex would be present and functional in the parasite (Additional Files 1 and 2). A mammalian deadenylase, PARN, was also missing from *Giardia*. Of the other parasitic protists examined to date for deadenylation machinery, *Trypanosoma brucei *has been shown to contain both the Ccr4 -Not complex and the Pan2-Pan3 complex [1,26], while *Plasmodium falciparum *contained a majority of the required components of the Ccr4-Not complex but was also missing the Pan2-Pan3 complex [27].

#### Decapping machinery

All parasitic protists examined to date contain traditional 5' 7-methylguanosine caps, although a biochemically modified cap structure is found on the spliced leader RNA in *Trypanosoma*. In *Giardia *the presence of 5' 7-methylguanosine caps have been identified on both coding and sterile transcripts [25]. The removal of this cap is required for 5'-3' exonuclease processing. Decapping in other cells requires the decapping holoenzyme, comprised of Dcp1p and the catalytic subunit Dcp2p. A wide array of other machinery has been shown to interact with and mediate the activity of Dcp *in vitro *and *in vivo*, including Lsm (like-Sm) proteins, the Upf proteins, the Edc proteins, Dhh1p and Pat1p; while more recent research has suggested the presence of additional members of the decapping family, such as *DcpS *and *Headless*, and an astonishing diversity of proteins associated with mRNA storage and decay (e.g. TTP/Brf1 and 2; AUF1; HuR; KSRP; CUG-BP) in specific lineages, making it clear that the process of mRNA decay is both precise and organism-specific [43,44]. These proteins and the decapped mRNA often assemble into degradation complexes referred to as P-bodies (processing bodies).

Here we identify a Dhh1p and a divergent *Giardia *Dcp2-like protein (Additional Files 1 and 2). Like many eukaryotic helicases, Dhh1p protein possesses a DEXDc superfamily domain and when reciprocally BLAST against *S. cerevisiae*, the top identified gene is the *Dhh1*. The *S. cerevisae *Dcp2 protein has both dcp2 and nudix domains. The dcp2 domain aids in mRNA cap removal while the nudix hydrolase domain catalyzes catabolism of nucleotide diphosphates linked to other molecules. The identified *Giardia *protein contains a nudix hydrolase domain but is missing the dcp2 domain. However, when used as a query in BLASTp, significant hits identify it as a likely mRNA decapping enzyme and a possible Dcp2 homolog. We were unable to identify the Edc proteins, Pat1p, or any of the organism-specific alternative decapping machinery and P-body components, such as *DcpS*, *Headless, TTP/Brf1and2. AUF1*, etc. We think it likely therefore that the evolutionary divergence of *Giardia *has dictated either a unique array of P-body proteins specific to the Diplomond lineage or perhaps P-bodies are simply absent from *Giardia*.

Another set of proteins which have roles in decapping and mRNA degradation are the Lsm (like-Sm) proteins. Members of the Sm/Lsm protein family are conserved in bacteria and archaea [45]. This family of proteins plays roles in RNA processing, splicing and mRNA decapping [46,47] and some of the proteins colocalize with the mRNA decapping machinery [48]. The Lsm complex involved in mRNA degradation is made of Lsm proteins 1-7 and they form a heteroheptameric ring. In total we were able to identify 14 proteins with Sm-like domains using the Interpro domain accessory function in the *Giardia *genome database (Additional File 2). Of the 14 proteins, 12 had Conserved Domain Database (CDD) recognizable Lsm domains, yet only four of these proteins possess notable sequence similarity to Lsm proteins in *S. cerevisiae*. The remaining 8 proteins are likely members of the Sm protein family which have roles in pre-mRNA splicing [46]. The results we see here are consistent with what is seen in the other complexes which we observed, in that some machinery is present and recognizable while other components cannot be identified. The homologous complex in bacteria, known as the Hfq complex is a homohexamer [45], therefore possibilities exist that Lsm complex may still form in *Giardia *despite only identifying four Lsm proteins.

### 5' to 3' Degradation

Once mRNA transcripts are decapped, they are accessible to the 5' to 3' degradation machinery. The proteins responsible for 5' to 3' degradation of eukaryotic mRNAs are the Xrn proteins [6,49]. *S. cerevisiae *possesses two Xrn proteins: Rat1p (nuclear) and Xrn1p (cytoplasmic). The nuclear form is responsible for nuclear processing of RNAs while Xrn1p is responsible for degradation of cytoplasmic mRNA transcripts. The XRN domain is located in the N-terminus of the protein and provides 5' to 3' exonucleolytic activity. Although *S. cerevisiae *has two homologs, *T. brucei *possesses four Xrnp homologs [33]. All four are expressed in *T. brucei*, although only two of the four are needed for growth. In our search, we were able to identify two proteins with sequence similarity to Xrnp in *Giardia *(GL50803_24133 and GL50803_113365) (Additional Files 1 and 2). The former is more similar to *S. cerevisiae *Rat1p whereas the latter has more similarity with Xrn1p. The presence of these two exonucleases supports the likely presence of a functional 5' to 3' degradation pathway.

### 3' to 5' Degradation

#### Identification of exosome components

Eleven main proteins comprise the eukaryotic exosome: six RNase PH proteins (Rrp41p, Rrp42p, Rrp43p, Rrp45p, Rrp46p, and Mtr3p), three stabilizing proteins (Rrp4p, Rrp40p and Csl4p), and two peripheral proteins (Rrp6p and Rrp44p). Rrp6p and Rrp44p are not always found in the exosome complex together within the same organism. For example, *S. cerevisiae *Rrp6p and Rrp44p are found only with the nuclear exosome and in both nuclear and cytoplasmic exosomes, respectively [13,14], while human Rrp6, and Rrp44 proteins (hDIS3 and hDIS3L) are localized to the nucleolus, the nucleus and cytoplasm, and primarily the cytoplasm respectively [50]. Human DIS3 and DIS3L display a lower affinity for the exosome than the *S. cerevisiae *homolog. In *S. cerevisiae*, *H. sapiens*, and *A. thaliana*, we found all eleven of the previously identified exosome genes, validating our similarity search protocol. However, in our investigation of the parasitic protists, we were unable to identify some specific exosome components. We first discuss our detailed characterization of the putative exosome proteins in *Giardia*, followed by our characterization of the exosome protein components in the other parasitic protists.

In the search for exosome components in *Giardia*, we had differential success depending on whether we used the PFAM or the CDD definitions. Using multiple approaches we identified only four of the expected six RNase PH domain proteins (Additional Files 1 and 2). GL50803_1890, GL50803_5632, and GL50803_40007 all contain RNase PH domains; GL50803_9847 does not have a true RNase PH domain but possesses the CDD multi-domain COG2123 that is associated with RNase PH domains (Additional File 1). It is unclear what arrangement of the four RNase PH domain proteins comprises the hexameric exosome in *Giardia*. Continuing this theme of genomic diminution, we were able to identify genes for only two of the three associated proteins, *rrp4 *and *rrp40 *but not *csl4*. We were also able to identify a gene for only one of the two exosome peripheral proteins: *rrp44*, identifiable by its RNase II-like domain (RNB), but not *rrp6*, or any other DNA Q domain-containing proteins. In addition to the putative Rrp44p homolog, we also were able to identify a second protein (GL50803_9912) containing an RNase II like domain in *Giardia*, but it displayed a sufficiently low similarity with Rrp44p that we cannot confirm a possible identity. The schematics in Additional File 1 provide a visual representation of the identified proteins in *Giardia *and their similarity to *S. cerevisiae *RNA degradation proteins.

In the other parasitic protists, *T. brucei*, *P. falciparum, T. vaginalis*, and *E. histolytica*, the identified protein repertoires for exosomes are more complete but often still partial (Table 1). *T. brucei *and *E. histolytica *had six proteins containing RNase PH domains, the correct number to construct a heterohexameric exosome. We note that our ability to find all six RNase PH domain proteins in *T. brucei *by similarity searches represents an improvement compared to previous studies [22,24] and indicates a high level of sensitivity for divergent protein sequences in our study. *T. vaginalis *had seven identifiable proteins containing RNase PH domains; this number more than accounts for enough proteins to form the hexameric exosome ring and reflects a common theme of gene family expansion in *T. vaginalis *[51]. However, *P. falciparum*, like *Giardia*, has only four proteins with recognizable RNase PH domains. Previous work from the DeRisi group was able to identify only three RNase PH proteins in *P. falciparum*. That work was published in 2007, where genome annotation was less complete than when we performed our search. They performed BLASTP and reciprocal BLASTP to identify the putative degradation proteins in *Plasmodium *[27].

**Table 1:** Exosome components in the parasitic protists

Core and peripheral eukaryotic exosome components									
	***S. cerevisae***	***H. sapiens***	***A. thaliana***	***T. brucei *(TREU)**	***P. falciparum***	***T. vaginalis***	***E. histolyica*** **(HM-1_IMSS)**	***G. lamblia *(WB)**	**Canonical Archaeal exosome**

**Ring Components**									
**rrp41**	YGR195W	*NP_061910	*AT3G61620	Tb927.10.7450					*NP_342241
**rrp42**	YDL111C	NP_055819		Tb927.1.2580					NP_342240
**rrp43**	YCR035C	NP_852480		Tb11.01.8320					**
**rrp45**	YDR280W	NP_001029366 NP_005024	**AT4G27490 AT3G46210 AT3G07750 AT3G12990 AT3G60500 AT1G60080**	Tb927.6.670	PF14_0256 PFB0415c MAL13P1.204 PF13_0340	**TVAG_250040 TVAG_441560 TVAG_287740 TVAG_027130 TVAG_453750 TVAG_189430 TVAG_220800**	**EHI_040320 EHI_166910 EHI_126330 EHI_086520 EHI_188080 EHI_000580**	**GL50803_ 1890 GL50803_5632 GL50803_40007 GL50803_ 9847**	**
**rrp46**	YGR095C	NP_064543.3		Tb927.2.2180					**
**mtr3**	YGR158C	NP_478126		Tb11.01.2820					**

**Stabilizers**									
**rrp4**	YHR069C	NP_055100	*AT1G03360	*Tb927.7.4670	PFD0515w	TVAG_246740	EHI_163510	GL50803_33022	NP_342242
**rrp40**	YOL142W	NP_057126	AT2G25355 AT4G32175	Tb09.160.5160	MAL13P1.36	TVAG_380110	EHI_004770	GL50803_17091	**
**csl4**	YNL232W	NP_057130	AT5G38890	Tb927.5.1200	MAL7P1.104	TVAG_110240 TVAG_121320	U	U	NP_341842

**Peripheral**									
**rrp6**	*YOR001W	*NP_001001998 NP_002676	AT5G35910 AT2G32415 AT1G54440	Tb927.4.1630	MAL13P1.311 PF14_0473	TVAG_053630 TVAG_197890 TVAG_283650	EHI_021400 EHI_064630	U	**
**rrp44**	*YOL021C	NP_001121698 NP_055768	AT2G17510 AT1G77680 AT5G02250	Tb11.02.5380 Tb11.01.0260	MAL13P1.289	TVAG_311220	EHI_160720	GL50803_112718	**

The chart displays a selection of organisms and the components of their exosomes divided into the ring, stabilizing and peripheral components. Representative metazoan eukaryotes, *S. cerevisiae*, *H. sapiens*, and *A. thaliana*, are identified on the left most side of the chart, while several species of single-celled eukaryotic parasites are listed in the middle and right side of the chart. The far right column displays the typical proteins composing an archaeal exosome. Components of the exosome whose activity has been confirmed experimentally are marked with asterisks. Gene annotations are from Saccharmoyces Genome Database (*S. cerevisiae*), National Center for Biotechnology Information (*H. sapiens sapiens *and canonical archaeal exosome *S. solfataricus*), The Arabidopsis Information Resource (*A. thaliana*), and Eukaryotic Pathogen Database (*T. brucei*, *P. falciparum*, *T. vaginalis*, *E. histolytica*, and *G. lamblia*). Boxes are coded as follows; normal font/identified, **bold font**/unable to identify the exact identity, **/non-existant, and U/not identified.

Additionally, *T. brucei*, *P. falciparum*, and *T. vaginalis *possess all three exosome stabilizing proteins, whereas *E. histolytica *like *Giardia*, has only *rrp4 *and *rrp40*, but lacks a *csl4 *homolog (Table 1). Unlike *Giardia *all four of these parasitic protists contained putative homologs for both *rrp6 *and *rrp44*.

#### Exonucleolytic potential of RNase PH and Rrp44 homologous proteins

To define the exonucleolytic potential of the degenerate *Giardia *exosome, we first sought to classify the RNase PH proteins as either Rrp41-like or Rrp42-like. The archaeal exosome is composed of three Rrp41-42 heterodimers, and the exonucleolytic activity is maintained solely in the Rrp41p subunit [13,52]. In eukaryotic species the exosome is composed of three Rrp41-like and three Rrp42-like proteins, although the exonucleoytic activity of the Rrp41-like proteins is sometimes lost due to mutation of the catalytic or the phosphate binding sites [8]. Since the eukaryotic exosome likely evolved from the archaeal exosome, we expected to be able to identify at least one Rrp41-like and Rrp42-like protein in each of the parasitic protists. With the goal of identifying whether the RNase PH proteins were Rrp41-like or Rrp42-like in their origin, we constructed protein phylogenies.

**Figure 1:**
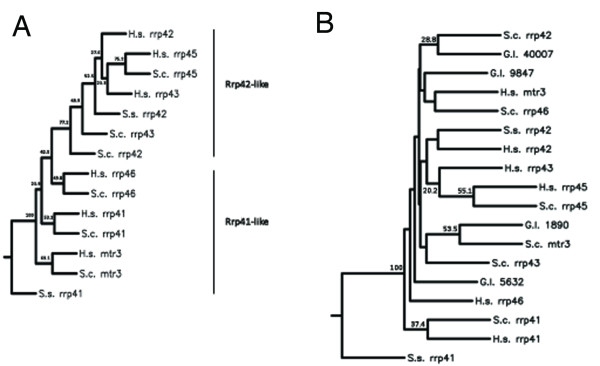
**Rnase PH protein phylogenies reveal an absence of two subunits in *Giardia***. Gene trees were made to diagram the relatedness of the RNase PH protein amino acid sequences in *S. serevisisae*, *H. sapiens*, *S. solfataricus*, and *G. lamblia*. Node values shown are out of 100 and nodes with no labels had values below 20. **(A) **The protein parsimony tree displays relatedness between RNase PH proteins of *S. cerevisiae*, *H. sapiens*, and *S. solfataricus*. The tree is separated into Rrp41-like and Rrp42-like proteins with node values providing moderate support for the inferred clades. **(B) **Addition of putative *Giardia *RNase PH proteins results in disrupted clades and reduced bootstrap values.

Our first step was construction of a protein phylogeny using Rrp41p-like and Rrp42p-like amino acid sequences from *S. cerevisiae*, *H. sapiens*, and *S. solfataricus *RNase PH protein amino acid sequences. Protein sequences were aligned in MUSCLE and trimmed using G-blocks as stated in the methods to limit our analysis to the more highly conserved regions of the proteins. This resulted in protein fragments of 110-115 amino acids from proteins of ~240 and ~300 amino acids for Rrp41-like and Rrp42-like proteins, respectively, for use in construction of the phylogeny. The retained amino acid sequences contained the RNase PH domain, with approximately ten amino acids flanking each end. The Phylip 3.69 package was run iteratively to generate 100 trees for estimation of bootstrap values. We observed distinct stratification of Rrp41-like and Rrp42-like proteins; however, node values showed only moderate support for placement into each Rrp subtype (Figure 1A), indicating high sequence divergence of these proteins.

However, with the addition of *Giardia *sequences, this divergence was profoundly exacerbated, and we observed considerable movement of sequences from previously strong clades that drastically altered the tree topology, eroding the stratification of the Rrp41-like and Rrp42-like proteins (Figure 1B). This phenomenon was observed even with the addition of single *Giardia *sequences (data not shown). Our inability to group these RNase PH proteins was not specific to *Giardia*, as similar difficulties were encountered with the use of RNase PH proteins from the other parasitic protists. Although frustrated in our attempt to classify the RNase PH domain proteins, our inability to produce informative gene trees about protein identity argues that high levels of sequence divergence are tolerated in the proteins that comprise the exosome ring.

As an alternative approach to classification of *Giardia's *RNase PH domain-containing proteins, we sought to determine which of the proteins possess the necessary amino acid residues for phosphate binding and catalytic activity. The amino acid alignment of the four genes containing the *Giardia's *RNase PH domains shows that while all possess the necessary residues for activity of the catalytic site, only GL50803_5632 has even one of the two amino acids required for phosphate binding (not shown). This technique, while not helping to classify the *Giardia *RNase PH proteins, does suggest that all RNase PH domain-containing genes in *Giardia *are nucleolytically inactive.

**Figure 2:**
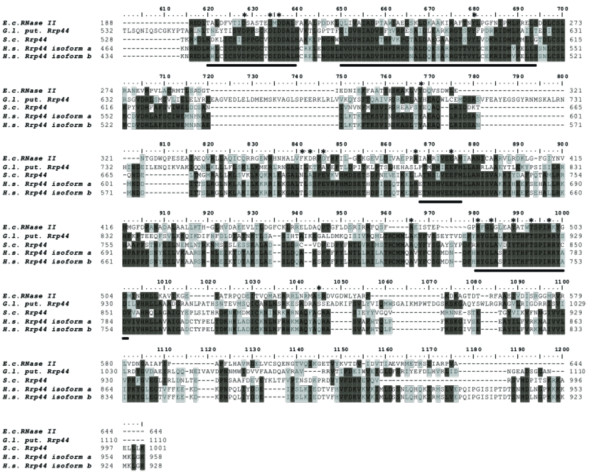
**Identification of Rrp44 protein catalytic potential in *Giardia***. Alignment of *E. coli *RNase II with Rrp44 amino acid sequences from *G. lamblia *(*G.l*.), *S. cerevisiae *(*S.c*.), and *H. sapiens *(*H.s*.) show levels of high conservation. Important regions required for RNA binding are underlined. Dark gray represents amino acid identity while the light gray represents amino acid similarity. The conserved amino acids in the catalytic pocket of *E. coli *RNase II are noted by an asterisk above the sequence.

In contrast, examination of the putative *Giardia *Rrp44 homolog revealed that this enzyme is most likely an active enzyme with exonucleolytic activity. When compared with *Escherichia coli *RNase II, *S. cerevisae *and *H. sapiens *Rrp44 proteins, the putative *Giardia *homolog shares a high percentage of catalytic residues present in the enzymatic pocket [12,53], although it is missing the classical PIN domain characteristic of some classes of nucleases [53] (Figure 2). The *E. coli RNase *II has 17 identified residues that are found in the catalytic site of the enzyme that are important for enzyme catalysis [12,53]. Human Rrp44p homologs possess 13 of the 17 residues found within the catalytic site [53], while *Giardia *and *S. cerevisiae *each possess 12 of the 17 amino acids. In one position in both *S. cerevisiae *and *Giardia *Rrp44 proteins, there is an amino acid substitution where a negatively charged amino acid is replaced with another negatively charged amino acid; *S. cerevisiae *has an aspartate in place of glutamate at D363 while *Giardia *has an aspartate in place of glutamate at D360. The substitution is not likely to result in alterations in bond forming. In addition, the putative *Giardia *Rrp44 homolog possesses all four of the domains believed to play a role in the RNA binding activity of the enzyme. These RNA binding domains are highly conserved in species as divergent as *H. sapiens *and *Giardia*. Based on the presence of these highly conserved domains, and the number of residues identified as part of the catalytic sites, we believe that it is likely that the putative *Giardia *Rrp44p homolog possesses exonucleolytic activity and thus the exosome in conjunction with putative Rrp44p in *Giardia *is also likely active. The absence of an Rrp6p homolog means that Rrp44p is potentially the only nucleolytically active enzyme in the *Giardia *exosome complex.

### Exosome-dependent complexes and proteins

#### TRAMP complex

The TRAMP complex is a nuclear-localized, exosome-associated protein complex, responsible for polyadenylation of RNA molecules that will be targeted for further processing or degradation [16,17,54]. The RNA binding proteins Air1p/2p are responsible for identification and binding of the targeted RNA transcripts. The poly-A polymerase-like proteins, Trf4p/5p are responsible for the addition of the poly-A tails to all RNA species that are incorrectly folded or aberrantly produced. Lastly Mtr4p, an RNA helicase, is thought to remove the secondary structure of folded transcripts. The Trf5p/Air1p/Mtr4p complex localizes mainly to the nucleolus, while the Trf4p/Air2p/Mtr4p complex is found throughout the remaining nucleus in *S. cerevisiae *[16].

We identified genes with sequence and domain similarity to *Trf4/5 *and *Mtr4 *in *Giardia *(Additional Files 1 and 2). The *Giardia *Trf4p/5p proteins contain the canonical nucleotidyltransferase domains, suggesting that they are functionally capable of adding poly-A tails to RNAs. However, we identified only one gene with a partial Air1 domain in the *Giardia *genome. *S. cerevisiae *proteins Air1p and Air2p both contain an Air1 domain, and deletion of either Air1p or 2p does not inhibit the processing of some snoRNAs and rRNAs, while deletion of both proteins leads to a dramatic increase in unprocessed snoRNAs [17]. Therefore, it is possible that both *Giardia *TRAMP complexes function normally in the presence of a single Air1 domain-containing protein.

#### Puf proteins

Members of the Puf family of proteins are identified in a variety of eukaryotes with a variable number of genes in each species [55-57]; different members of the Puf protein family are either nuclear or cytoplasmic in their localization. Although in some cell types, Puf proteins act to stabilize mRNA transcripts and increase levels of translation, more typically Puf proteins reduce mRNA expression either through inhibition of translation or through mediation of mRNA decay. For example, Puf5 in yeast binds to Pop2 (Caf1) to recruit the Ccr4p deadenylase to the 3' UTRs of mRNAs in the cytoplasm [18,19]. Given the unusually short 3' UTRs of *Giardia *mRNAs, it was not apparent that the Puf proteins would be relevant in the parasite, but we were able to identify a full repertoire of five genes containing Puf repeats in *Giardia*. Higher eukaryotes generally have fewer members of the family [56-58]. Puf proteins normally possess eight Puf repeats in the C-terminus [56], although studies in *S. cerevisiae *suggest that six Puf repeats are sufficient for RNA binding [59]. Of the five identified proteins in *Giardia*, one contains eight Puf repeats, three contain between five and seven repeats in the C-terminal half of the protein, and one may be a pseudogene with only a partial N-terminal RNA binding domain with only three repeats (Additional Files 1 and 2). Amino acid sequence comparison with *S. cerevisiae *Puf protein sequences suggests that the putative *Giardia *Puf proteins may be homologs of *S. cerevisae *Puf proteins that primarily bind ribosomal RNAs and mRNAs encoding nuclear localized proteins. This designation was obtained using *Giardia *Puf proteins as query for BLASTP in the *Saccharomyces *genome database.

#### Nonsense-mediated decay

Nonsense-mediated decay (NMD) is responsible for the translation-coupled degradation of mRNA transcripts containing premature termination codons (PTCs) [60,61], Yet, while PTCs are defined as any stop codon upstream of an exon junction complex, NMD is functional in single exon transcripts. Thus, while stop codons upstream of exon junction complexes may trigger the NMD mechanism, they are not the only stimulus. An alternative explanation is that the distance between the stop codon and the poly-A binding protein (PABP) is what triggers NMD, such that increasing the distance between the stop codon and the PABP increases the chance of NMD activation.

The NMD complex has been previously shown to be functional in *Giardia*, although the precise means by which transcripts lacking introns in *Giardia *are targeted for degradation by the pathway has yet to be identified [28,42]. These studies by Sun and colleagues identified 7 of 14 NMD associated factors by sequence similarity. The putative homologs of the following NMD genes in *Giardia *were identified: *upf1*, *eRF1 *and *eRF3*, *sMG1*, *hrp1*, *xrn1 *and *xrn2*, and *ski7 *(Additional File 2) [28].

**Figure 3:**
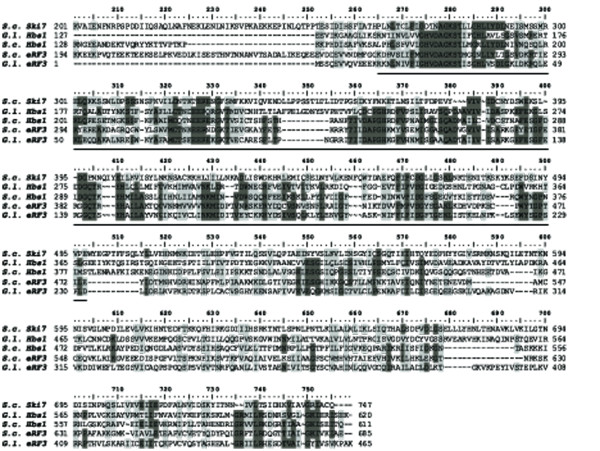
**Identification of EF1 family proteins in *Giardia***. Alignment of members of the EF1 family from *G. lamblia *(*G.l*.) and *S. cerevisiae *(*S.c*.). Important regions required for RNA binding are underlined. Dark gray represents amino acid identity while the light gray represents amino acid similarity. The GTPase domains common to members of the family are underlined. The Hbs1 and eRF3 homologs are on average twice as similar to each other than they are to Ski7.

eRF1p and eRF3p are eukaryotic release factors that function in normal translation termination to remove the ribosome from the mRNA [62]. The proteins Ef1αp, eRF3p, Hbs1p, and Ski7p belong to the EF1 family, and all possess elongation factor GTP binding domains (Additional File 1). We believe that the proposed Ski7p homolog was incorrectly identified by Sun and colleagues and is actually the *Giardia *Hbs1p homolog. Amino acid alignments indicate that the *Giardia *Hbs1 protein sequence is approximately twice as similar to the *S. cerevisiae *eRF3 protein sequence as it is to the *S. cerevisiae *Ski7 protein sequence (Figure 3). Additionally, Hbs1 proteins possess two translation factor domains, both of which are absent from Ski7 (Additional File 1) and are present in the proposed *Giardia *Ski7p protein. With the alignment data, domain identification, and with the knowledge that *ski7 *has only previously been found in some *Saccharomyces *species [62], we believe that the gene previously identified at *ski7 *is actually *hbs1*. However, the relationship between the members of the protein family make it likely that Ski7p and other EF1αp homologs may be interchangeable in their functionality in different eukaryotes, so that even without Ski7p, mRNA quality control pathways such as NMD and NSD may still remain functional [62].

#### Non-stop decay

Acting as a counterpoint to the NMD mechanism of RNA degradation, and sharing some of the same protein machinery, non-stop decay (NSD) is again translation-dependent but is alternatively activated when the ribosome fails to encounter a stop codon during translation of an mRNA transcript [63,64]. In *S. cerevisiae*, the ribosome continues reading into the poly-A tail and stalls at the end of the transcript, followed by recruitment of Ski7p - and possibly other proteins - to the ribosome. Given that Ski7p has only been identified in a subset of *Saccharomyces *species, and a NSD-like mechanism has been functionally identified in HeLa cells with the demonstration that an mRNA transcript lacking a stop codon is degraded more quickly than the same transcript with a stop codon [64], it seems likely that machinery beyond Ski7 must be involved, although the HeLa cell machinery responsible for nonstop transcript degradation has not been identified. In *Giardia *we identified proteins with sequence and domain similarity to eRF3p and Hbs1p along with two identical Ef1α homologs. Since these proteins all share functional domains with Ski7p (Additional Files 1 and 2), they may possibly be able to trigger a NSD like mechanism in the absence of Ski7p [62,64].

#### No-go decay

Like NMD and NSD, No-go decay (NGD) is also translation-dependent, but it is triggered when the ribosome stalls upon encountering secondary structure in an mRNA transcript. Stalling of the ribosome due to other mRNA sequence-related factors can also induce NGD [65]. The stalling of the ribosome causes the recruitment of Dom34p and Hbs1p; Dom34p is a homolog of eRF1p, while Hbs1p is a homolog of eRF3p; Endonucleolytic cleavage ensues and the two halves of the mRNA transcript are degraded. *Giardia *possesses proteins with sequence and domain similarity to both Dom34p and Hbs1p (Additional Files 1 and 2).

### RNA interference machinery

The RNAi machinery consists of Dicer and Argonaute proteins. Dicer possesses single Paz and RNase III domains [66-68]. The guide strand of the dsRNA is loaded into an Argonaute homolog that forms the RNA-induced silencing complex (RISC) [66,67]. Argonaute homologs contain one Piwi and one Paz domain each [68]; the Piwi domain binds the RNA at its 3' end, while the PAZ domain possesses a nuclease-like activity that cleaves or inhibits translation of the bound target mRNA transcript. In some organisms, the dsRNA signal can be amplified through the use of an RNA dependent RNA polymerase (RdRP), which uses the Dicer product as a primer for subsequent dsRNA synthesis.

RNAi has been documented in a broad range of eukaryotes such as *T. brucei, Drosophila melanogaster*, *H. sapiens*, and *Schizosaccharomyces pombe *with roles in gene silencing and heterochromatin formation [5,47]. However, RNAi machinery is absent from other eukaryotes such as *P*. *falciparum *and *S. cerevisiae *[68,69], and in *Toxoplasma gondii*, the RNAi machinery has resisted functional characterization [70]. *Giardia's *RNAi machinery has been previously well-characterized and consists of an RdRP, Dicer and Argonaute homologs [39,68,71] (Additional File 2). Knockdown of *Giardia *RdRP or Dicer results in altered gene expression, especially concerning the variant surface proteins (VSP) on the plasma membrane of trophozoites, while Argonaute is an essential gene [39]. The observation of different effects in *Giardia *depending on which gene in the RNAi pathway is knocked down, leads us to believe that some proteins in the pathway have roles in the cell beyond that of just RNAi.

## Discussion

The importance of RNA degradation is evident from the multitude of pathways and mechanisms present in eukaryotic cells. RNA transcripts must be degraded to remove both non-functional transcripts and transcripts that are no longer needed for translation. Indeed, given the recent discovery of the ubiquitous nature of transcription along eukaryotic genomes [72], there is a rising appreciation for the role of RNA degradation in the regulation of gene expression. The exosome serves as a basic machinery of RNA degradation in eukaryotic cells, but the complex does not act alone. Additional complexes such as TRAMP in the nucleus and NSD, NMD, and NGD in the cytoplasm assist the exosome with degradation of mRNA transcripts. Furthermore, complexes such as the RNAi pathway degrade dsRNA into shorter transcripts that are used for gene regulation and are not dependent on the exosome for their function. The diversity of these complexes allows complete degradation of all RNA species found in the cell.

In this study we used a bioinformatics approach to investigate the exosome of the parasitic protists with a special focus on the RNA degradation machinery of *Giardia lamblia*. Although our analyses described here specified only the *Giardia *Genome isolate, we found that *Giardia *isolates P15 and GS possessed the same exosome components as the Genome strain (data not shown). We were able to identify several putative components of *Giardia's *exosome, including four RNase PH proteins for the ring of the exosome; Rrp4 and Rrp40, as stabilizing proteins for the ring structure; and lastly an Rrp44 homolog that we predict possesses the requisite nucleolytic activity. There are no trends in the expression patterns of proteins from the different complexes, when looking at normal growth and development, and also stress responses of *Giardia*.

One of the most striking results of our research is the detection of only four RNase PH proteins. Our findings expand the repertoire of putative RNase PH proteins beyond the two (A8BNT9/GL50803_1890 and C6LWS9/GL50803_5632) identified through a bioinformatics analysis in a recent publication from Clayton and colleagues [73]. The other paper did not define their search parameters in detail, and we suspect that the difference in our findings is the consequence of more relaxed search parameters in the searches described in this paper. We stress, however, that all four putative RNase PH proteins passed a reciprocal BLASTp search test and contained either strong consensus PH domains, or, in the case of GL50803_9847, the more generic, but related COG2123 domain. Likewise, we identified four putative RNase PH proteins in *Plasmodium falciparum*, one more than DeRisi and colleagues identified [27], although the fourth one has subsequently been identified in online databases. Given the extreme sequence divergence that often characterizes proteins in the parasitic protists compared to other eukaryotes, it is possible that an *in silico *approach is inadequate to identify the full protein family. Indeed, this proved to be the case in earlier studies in *T. brucei, P. falciparum*, and *C. reinhardti *[24,27,74]. However, our search algorithm was capable of detecting all six RNase PH domain proteins in *T. brucei*, suggesting an enhanced sensitivity relative to previous efforts. Thus, our inability to identify all exosome components in *Giardia *and *Plasmodium *that were previously identified in *S. cerevisiae *and *H. sapiens *is likely either the result of true gene absence or sequence divergence of the unidentified proteins. The *Giardia *genome sequencing project has 11× coverage of the genome with an estimated 96% of the genome sequenced [36], while the *P. falciparum *sequencing project has a minimum of 9× coverage for its chromosomes [75], further reducing the likelihood that genes could be missed because they are not yet part of the genome database

If *Giardia *and *Plasmodium *are indeed limited to four RNase PH proteins, this would mark a departure from the typical eukaryotic exosome structure and suggests that these proteins must be present in multiple copies within the exosome ring in order for *Giardia *and *Plasmodium *to have functional hexameric exosomes. We believe it unlikely that *Giardia's *and *P. falciparum's *exosomes differ this dramatically from all other known exosome structures in eukaryotes and archaea and from bacterial PNPase by having fewer than six RNase PH subunits building the exosome ring structure. The hexameric structure has been conserved in all domains of life and is still the most likely structure for *Giardia *and *Plasmodium*. Studies involving *C. reinhardtii *demonstrate that absence of *mtr3 *exosome component may be tolerated in some eukaryotes [74], providing some precedent to the scenario presented here. Therefore, one central finding from our research is that the core structure of the eukaryotic exosome may not be universal, and the exosome may be able to be composed of different combinations of Rrp41-like and Rrp42-like proteins, with potential for different stoichiometric ratios of the subunits. Unfortunately, the extreme sequence divergence of the parasitic protist RNase PH domain proteins interferes with our ability to accurately classify them, and we can therefore not be more specific in our description of the construction of the *Giardia *and *Plasmodium *altered exosomes.

Additionally, our inability to identify a *csl4 *homolog is initially striking since the archaeal exosome possesses a *csl4 *homolog, and *S. cerevisiae *is nonviable in *csl4 *deletion mutants [9]. However, *T. brucei *conditional mutants survive, with slowed growth rates, in the absence of the Csl4, although exosome functionality was never determined in the *T. brucei *Csl4 conditional mutants [24]. Tolerance of deleted genes is likely different in every organism but because Rrp4, Rrp40 and Csl4 all possess S1-like domains, it seems likely that Rrp4 or Rrp40 may be able to act as a replacement for Csl4.

Perhaps most surprisingly, while we were able to identify a likely Rrp44 homolog, we did not identify an Rrp6, or indeed any RNase D-like, homolog. The absence of Rrp6 appears to be specific only to *Giardia *since it was found in the other parasitic protists we examined. Similar studies of *C. reinhardtii *were also unable to identify an Rrp6 homolog, but they were able to detect several putative open reading frames with homology to RNase D domains [74] that likely serve as divergent substitutes. In *S. cerevisiae*, Rrp6 is nuclear localized and acts to degrade unstructured RNAs. In *S. cerevisiae*, deletion of Rrp6 is tolerated, but cells display steady state increases in RNA transcripts such as antisense and cryptic unstable transcripts (CUTs) [76,77]. These transcripts are normally produced but fail to accumulate to significant levels because they are degraded by Rrp6 [76].

The absence of an Rrp6 homolog in *Giardia *is intriguing in light of the abundance of stable noncoding antisense transcripts in the parasite [37,38,78]. The Nab3 and Nrd1 proteins were also unidentified. These proteins play roles in maintaining undetectable levels of CUTs in *S. cerevisiae *[76,77]. CUTs and *Giardia *antisense transcripts are similar because both can be produced from cryptic promoter sequences in the genome or from bidirectional transcription at defined promoters [38,76,77,79]. However, *Giardia's *antisense transcripts are quite different from CUTs. CUTs are capped at their 5' ends and are approximately 300 nucleotides or shorter in length [77]. Whereas *Giardia *antisense transcripts are also capped at their 5'end, they can be thousands of nucleotides long and are polyadenyated [25,37]. Perhaps, most importantly, *Giardia's *antisense transcripts are apparently stable, long-lived transcripts [37,78] and therefore, while cryptic, cannot accurately be termed CUTs. We suggest that the absence of *Giardia's *Rrp6 and other proteins such as Nrd1 and Nab3 [80,81] may play a role in the relative stability of these antisense transcripts, an observation for which we have previously been unable to provide a molecular explanation.

If the exosome is independently incapable of recognizing and degrading sterile transcripts in *Giardia*, we need to look to the other exosome-dependent and exosome-independent machineries to serve this role. In particular, we focus our attention on the cytoplasmic RNA degradation machinery since recent evidence has shown that antisense transcripts are exported from the nucleus into the cytoplasm in *Giardia *(Teodorovic and Elmendorf, in prep.). The non-exosome RNA quality control machinery is also ancient, with homologs in archaea: an EF1α homolog (aEF1A), an eRF1 homolog (aRF1), and an eRF1 family member (aDom34) [62,82,83]. Eukaryotes possess an expanded EF1 family consisting of EF1α, eRF3, Hbs1, and in some *Saccharomyces *species, Ski7. Interpreting the function of this machinery is complicated, however, since most of the protein components have dual roles in translation and in translation-dependent RNA quality control pathways. Thus, although *Giardia *possesses a full complement of these proteins, other than Ski7, we can't yet conclude that NSD and/or NGD are functional within the parasite.

## Conclusion

The key finding from this study is the identification of RNA degradation pathways in a group of highly divergent eukaryotes, the parasitic protists. These protists display an unusual reluctance to regulate gene expression at the transcriptional level, instead often engaging in promiscuous transcription and reliance on post-transcriptional regulation of gene expression. A better understanding of the RNA degradation machinery in these parasitic protists provides us with the opportunity to answer intriguing basic molecular biology questions about the range of structural variation that is permissible in a functional eukaryotic exosome. Our research has revealed the surprising finding that the 'canonical' eukaryotic exosome composition of six different RNasePH domain-containing proteins (three Rrp41-like and three Rrp42-like subunits) is not universal, and both *Giardia *and *Plasmodium *each contain only four identifiable RNasePH domain-containing proteins, while *Trichomonas *contains a record seventh protein. While a theme of 'genomic minimalism' has been identified previously in *Giardia*, our findings presented here emphasize the extreme nature of this mechanistic diminution.

Our research also helps us to understand the unusual status of ncRNAs in *Giardia*. Our laboratory has previously published on the abundance and atypical stability of long ncRNA transcripts in the parasite. While we have now understood their origins for several years, we have previously been unable to explain their stability. Our detailed examination of the exosome in *Giardia *reveals the absence of Rrp6 and Nab3-Nrd1, and that difference may at last provide an explanation for this phenomenon.

## Methods

### Searching for degradation machinery of the RNA exosome

Sequence similarity and protein domain searches were performed to identify potential RNA degradation machinery components of the exosome in *Giardia lamblia *(WB), *Entamoeba histolytica *(HM-1:IMSS), and *Trichomonas vaginalis *(G3). Parallel searches were performed on organisms with previously identified exosome machinery (e.g. *Trypanosoma brucei *(TREU), *P. falciparum *(3D7), *Arabidopsis thaliana, and Homo sapiens*) to validate the efficacy of our search protocol.

We used *S. cerevisiae *RNA degradation machinery protein sequences as queries in our searches because many of the yeast homologs have been verified functionally. When putative homologs could not be identified using *S. cerevisiae *sequences, we additionally used human and *T. brucei *RNA degradation machinery protein sequences as queries in our searches. We performed initial searches using the BLASTP algorithm with default search parameters against deprecated and accepted open reading frames (ORFs) in the parasite genomes available in the Eukaryotic Pathogen Database Resource (EupathDB.org). The default parameters were set according to Washington University BLASTP default parameters (cpus = 2, topcomboN = 1, V = 100, B = 20, hspmax = 1000000, gi E = 1e-3, wordmask = seg, hspsepQmax = 4000, span1). For genes that could not be identified using BLASTP alone, we used the Interpro domain accessory function in the *Giardia *genome database (GiardiaDB.org) to search the genome with PFAM domains or Conserved Domains that define the RNA degradation machinery components. The domains we searched for include the RNase PH (PF01138), AIR1 (COG5082), NOT1 (PF0454), NOT2_3_5 (PF04153), NOT3 (PF04065), Exo_endo_phosph (PF03372), S1 (PF00575), KH (PF00013), Hit-like (PF01230), DCPS (PF05652), DCP1 (PF06058), RCD (PF04078), and the DNAQ_like_exo_superfamily domains.

### Parameters for acceptance or refusal of queried results

When using BLASTP, acceptance of queried protein output was based on E-value (≤ 0.05) and the presence of conserved RNA-binding or exonucleolytic protein domains found in other eukaryotic homologs. For proteins that were not conclusively identified in the initial search, all 'hits', regardless of E-value, were examined for the presence of the conserved RNA-binding or exonucleolytic protein domains when compared to the query sequence. This flexible search protocol is often necessary in organisms as divergent as the parasitic protists examined here. Parasite sequences at this stage were finally validated in a reciprocal search against the *S. cerevisiae *genome at the *Saccharomyces *Genome Database (yeastgenome.org). Parasite genes that identified the initial *S. cerevisiae *search sequence as the top hit were accepted as correct. The proteins in the resulting list were categorized and assigned to protein complexes.

### Amino acid alignments

All amino acid alignments were made using MUSCLE 3.8.31 [84] (parameters: gapopen -12.0, gapextend -1.0, center at 0.0). The RNase PH protein alignments were performed using a BLOSUM45 matrix, while all other alignments were made using default parameters (PAM 200 scoring matrix). G-Blocks was used to exclude portions of the RNase PH amino acid sequences with limited sequence similarity beyond the RNase PH domain [85,86]. G-Blocks settings were always set to the default parameters except for maximum number of contiguous non-conserved positions (10), minimum block length (4), allowed gap positions (with half), and use of similarity matrices (yes). All alignments are displayed using Bioedit Sequence Alignment Editor graphic display [87].

### Protein Phylogenies

Amino acid alignments of the RNase PH proteins from MUSCLE were used in the Phylip-3.69 package for the production of gene trees. All programs mentioned below are part of the Phylip-3.69 package [88]. Seqboot was used to bootstrap the data set 100 times with block size set at 1 (regular boostrap). Next, Protpars was used to infer relatedness of the sequences to one another from analysis of the 100 data sets produced from Seqboot. *Solfolobus solfataricus *Rrp41 was set as the outgroup. Lastly the freeware program Consense was used to produce the final gene tree. The consensus type used was extended majority rule. Extended majority rule allows groups of sequences that appear in more than 50% of trees to be included in the final tree and other sequences which fall below this level are then added until all sequences are accounted for.

## Authors' contributions

CW conceived of the study, performed the described bioinformatics and phylogenetic analyses, and drafted the manuscript. HE participated in its design and helped to draft the manuscript. All authors read and approved the final manuscript.

## Supplementary Material

Additional file 1 **Comparison of domain profiles in RNA degradation machinery between *Saccharomyces cerevisiae *and *Giardia lamblia***. This file is in PDF format and contains schematic representations of protein components of the RNA degradation machinery complexes. Functional domains and protein lengths are indicated in these schematics to allow a fuller understanding of the repertoire of RNA degradation machinery pathways in *Giardia lamblia*. Click here for file

Additional file 2 **Classification of the Putative RNA Degradation Machinery Protein Components in *Giardia lamblia***. This file is in Excel format and contains a table of the complete list of identified RNA degradation complexes and their protein constituents in *Giardia lamblia*. Click here for file
