# Supplementary material for: Identification and analysis of the RNA degrading complexes and machinery of Giardia lamblia using an in silico approach
Source: BMC Genomics. 2011 Nov 29;12:586. doi: 10.1186/1471-2164-12-586 (PMC3282835; doi:10.1186/1471-2164-12-586)
Supplement: Additional file 1 — Comparison of domain profiles in RNA degradation machinery between Saccharomyces cerevisiae and Giardia lamblia. This file is in PDF format and contains schematic representations of protein components of the RNA degradation machinery complexes. Functional domains and protein lengths are indicated in these schematics to allow a fuller understanding of the repertoire of RNA degradation machinery pathways in Giardia lamblia. [file 1471-2164-12-586-S1.DOCX]

***In silico* Approaches to Elucidating the RNA Degradation Machinery in *Giardia lamblia***

**Williams and Elmendorf**

**Additional File 1**

Supplemental Figure 1. Comparison of domain profiles in decapping and deadenylation proteins between *Saccharomyces cerevisiae* and *Giardia lamblia*. Domain overlap is shown by a second domain below the protein schematic. Truncated domains are denoted by an asterisk on the side where the domain is truncated. (A) Decapping and deadenylation proteins of *Saccharomyces cerevisiae*. (B) Proposed decapping and deadenylation proteins of *Giardia lamblia*.

Supplemental Figure 2. Comparison of domain profiles in 5’ to 3’ degradation machinery proteins between *Saccharomyces cerevisiae* and *Giardia lamblia*. Truncated domains are denoted by an asterisk on the side where the domain is truncated. 5’ to 3’ degradation machinery (B) Proposed 5’ to 3’ degradation machinery proteins of *Giardia lamblia*.

Supplemental Figure 3. Comparison of domain profiles in exosome proteins between *Saccharomyces cerevisiae* and *Giardia lamblia*. Truncated domains are denoted by an asterisk on the side where the domain is truncated. (A) Exosome proteins of *Saccharomyces cerevisiae*. (B) Proposed exosome proteins of *Giardia lamblia*.

Supplemental Figure 4. Comparison of domain profiles in TRAMP complex proteins between *Saccharomyces cerevisiae* and *Giardia lamblia*. Domain overlap is shown by a second domain below the protein schematic. Truncated domains are denoted by an asterisk on the side where the domain is truncated. (A) TRAMP complex proteins proteins of *Saccharomyces cerevisiae*. (B) Proposed TRAMP complex proteins proteins of *Giardia lamblia*.

Supplemental Figure 5. Comparison of domain profiles in PUF proteins between *Saccharomyces cerevisiae* and *Giardia lamblia*. Domain overlap is shown by a second domain below the protein schematic. Truncated domains are denoted by an asterisk on the side where the domain is truncated. (A) PUF proteins of *Saccharomyces cerevisiae*. (B) Proposed PUF proteins of *Giardia lamblia*.

Supplemental Figure 6. Comparison of domain profiles in NSD and NGD proteins between *Saccharomyces cerevisiae* and *Giardia lamblia*. Domain overlap is shown by a second domain below the protein schematic. Truncated domains are denoted by an asterisk on the side where the domain is truncated. (A) NSD and NGD proteins of *Saccharomyces cerevisiae*. (B) Proposed NSD and NGD proteins of *Giardia lamblia*. Proteins of NMD are not listed and can be seen in [28].

Supplemental Figure 1A.

Supplemental Figure 1B.

Supplemental Figure 2A.

Supplemental Figure 2B.

Supplemental Figure 3A.

Supplemental Figure 3B.

Supplemental Figure 4A.

Supplemental Figure 4B.

Supplemental Figure 5A.

Supplemental Figure 5B.

Supplemental Figure 6A.

Supplemental Figure 6B.
